# Acute cardiac tamponade following thoracoscopic lobectomy: a case report and literatures review

**DOI:** 10.1186/s13019-023-02374-3

**Published:** 2023-10-10

**Authors:** Wei Chen, Yi Shen, Yang Yuan, Qiangqiang Zheng, Yunfeng Zhou

**Affiliations:** Department of Thoracic Surgery, West China School of Public Health and West China Fourth Hospital, Sichuan University, Chengdu, 610044 Sichuan P.R. China

**Keywords:** Cardiac tamponade, Lobectomy, Pericardiocentesis, Pericardiotomy

## Abstract

Thoracoscopic lobectomy is a common surgical procedure for the treatment of lung cancer. With the continuous development of surgical techniques and medical devices, complications after thoracoscopic lobectomy are less and less, and cardiac tamponade is even rarer. This case is a 62-year-old woman who underwent thoracoscopic left upper lobectomy for a left upper lobe nodule. The patient developed acute cardiac tamponade on postoperative day 2, and symptoms resolved after pericardiocentesis. However, 20 h later, the patient underwent emergency surgery for re-developed acute cardiac tamponade, which was found to be a coronary tear. A review of the literature suggested that cardiac tamponade is more common in left lung surgery than right lung surgery. Pericardiocentesis can resolve initial acute cardiac tamponade, but pericardiotomy may be urgently needed after recurrence.

**Supplementary Information:**

The online version contains supplementary material available at 10.1186/s13019-023-02374-3.

## Introduction

Over the years, the incidence of complications and mortality following thoracoscopic lobectomy have been significantly reduced, but some unpredictable and even potentially life-threatening complications do occur. This report details a case of acute cardiac tamponade after thoracoscopic lobectomy and reviews the relevant literature to highlight potential causes and urgent interventions for the development of acute cardiac tamponade.

## Case presention

**Fig. 1:**
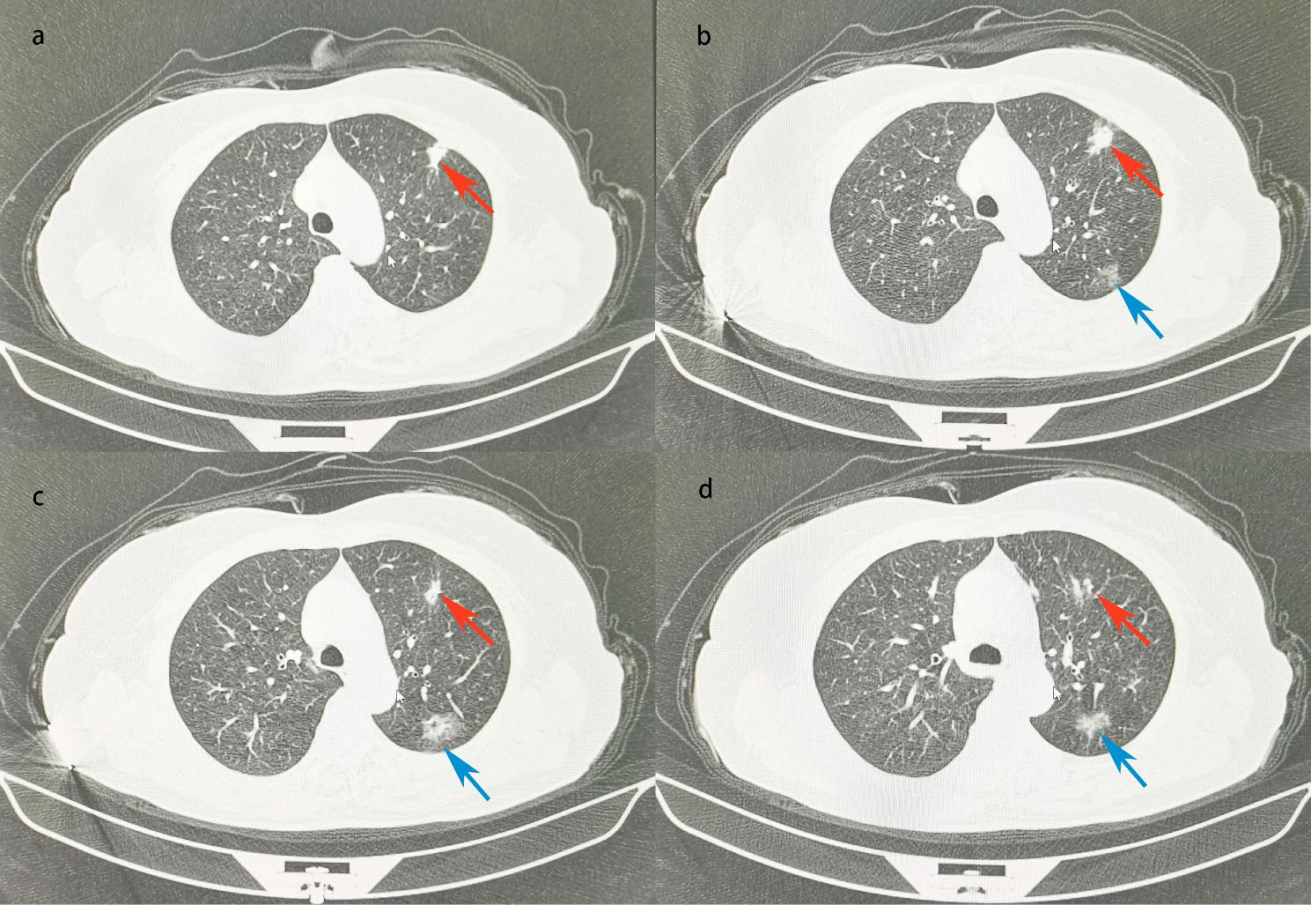
Chest CT showed an irregular solid nodule with a size of about 1.7 cm*1.1 cm in the S3 segment of the left lung (red arrow), and a mixed ground-glass nodule with a size of about 2.6 cm*1.8 cm in the S1 + 2 segment of the left lung (blue arrow)

**Fig. 2:**
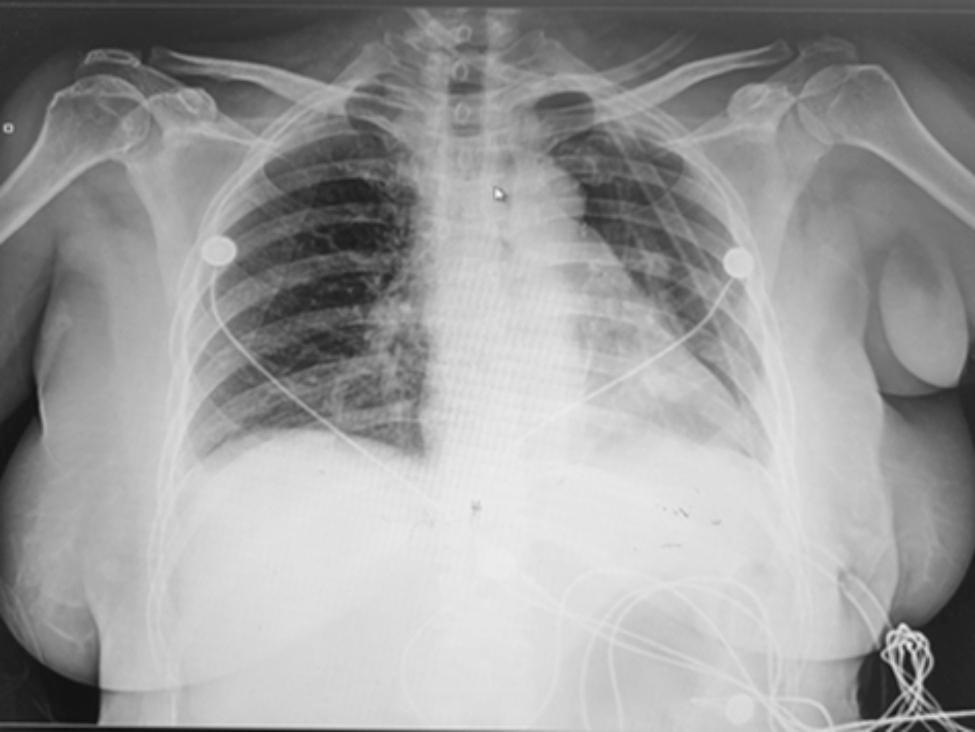
Postoperative chest X-ray showed good recruitment of the left lung without obvious pleural effusion

**Fig. 3:**
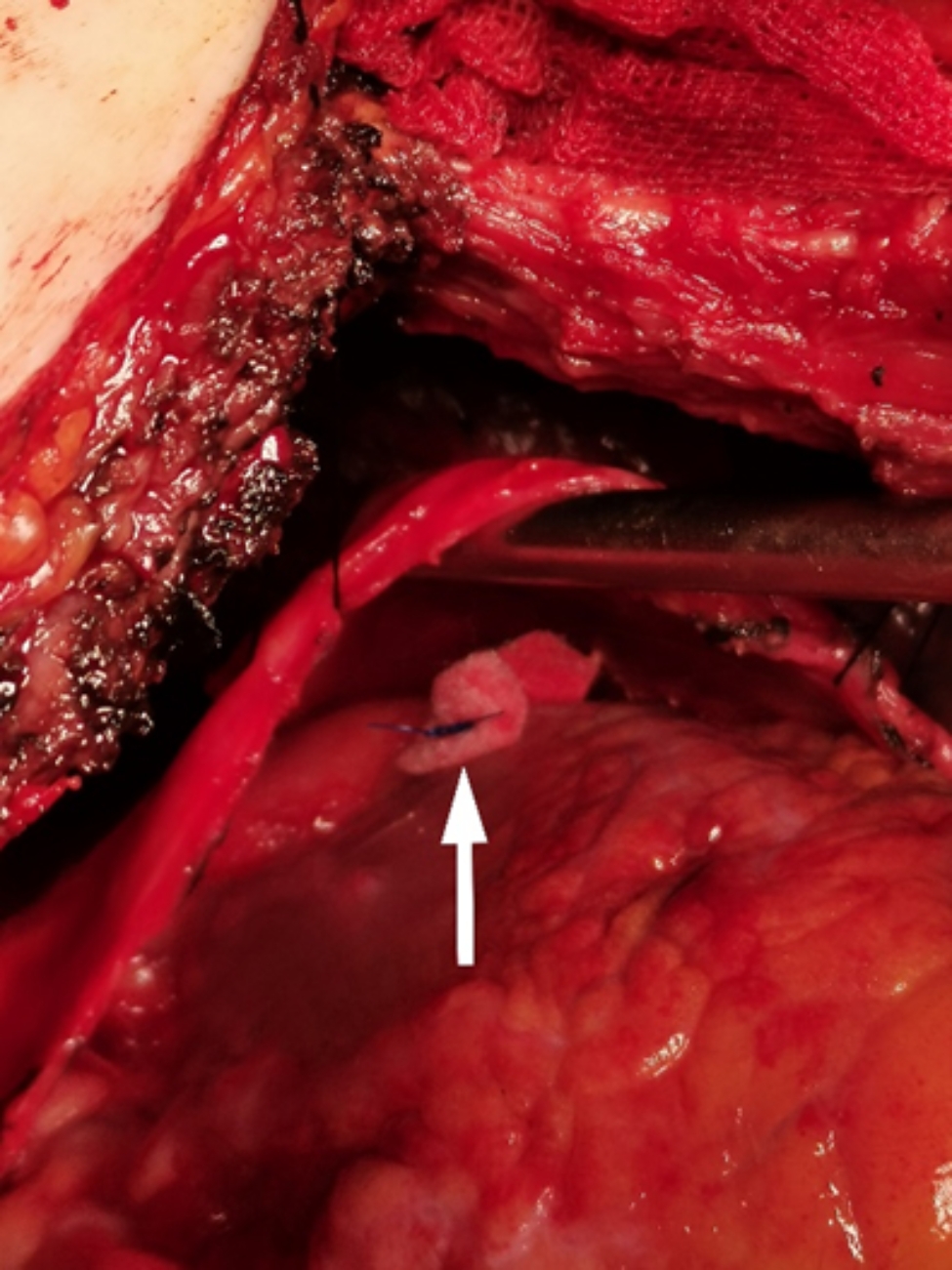
The sutured coronary laceration (white arrow)

A 62-year-old female was admitted to the hospital for the management of a " nodule of the left upper lobe " (Fig. 1). The patient had a 10-year history of diabetes treated with oral acarbose and metformin. Fasting blood glucose was elevated at 10.9mmol/L with a glycosylated hemoglobin (HbA1c%) of 11.1; subcutaneous insulin was injected for better glucose control. She had no history of hypertension or heart disease and denied cigarette or alcohol use. The patient’s cardiopulmonary function was assessed as satisfactory, pulmonary function showed that FEV1 was 2.04 L and FEV1/FVC was 81%, color doppler echocardiography showed that EF was 64%. The patient had no obvious surgical contraindications, so thoracoscopic left upper lobectomy with mediastinal lymph node dissection was performed. On the second postoperative day, the patient suddenly lost consciousness with hypotension and sinus tachycardia with a blood pressure of 73/46 mmHg and a heart rate of 131/min. Blood glucose was monitored at 9.4 mmol/L, and blood gas analysis showed metabolic acidosis. No myocardial ischemia was found on the electrocardiogram, and no abnormality was found in the blood routine and bedside chest X-ray (Fig. 2). After extensive fluid infusion and resuscitation, the patient’s consciousness improved, but She remains in shock and has vomiting and chest pain. Color Doppler echocardiography showed a collapsed right heart, and pericardial examination showed areas of dark fluid. Under ultrasound guidance, approximately 50 ml of non-coagulated blood was aspirated by pericardiocentesis. Subsequently, the patient regained consciousness and her vital signs returned to a stable state. However, 20 h later, the patient reappeared with vomiting, chest pain and disturbance of consciousness. Echocardiography confirmed recurrent cardiac tamponade. We performed emergency surgery, and exploration showed that the hilar stump was well closed without leakage, but the pericardium was distended and purplish red. After incision of the pericardium, 200 ml of non-coagulated blood was aspirated, and a thrombus was seen surrounding the heart and drainage tube. After removal of the thrombus, the heart wall was unremarkable, but a left circumflex coronary artery was found to have ruptured and continued bleeding, which we repaired with 4 − 0 Prolene sutures (Fig. 3).

The patient recovered well without subsequent complications and was discharged 13 days after pericardiotomy. Postoperative pathological examination revealed lung adenocarcinoma (pT1N0M0) and granuloma.

## Discussion

Acute cardiac tamponade is a very rarely encountered complication following lobectomy. Previously reported cases (Table 1) with known causes of pericardial tamponade following pneumonectomy are summarized as follows: (1) vascular stump retraction into the pericardium [1–3]; (2) direct pericardial injury, or injury to the ventricular wall or great vessel wall [4–7]; and (3) spontaneous coronary artery rupture [8]. Because the heart is located on the left side of the mediastinum, the pericardium has a larger contact area with the left thoracic cavity, and the left thoracic cavity is relatively narrow, we speculated that a left lung operation would have higher likelihood of damage to the pericardium and adjacent structures, resulting in cardiac tamponade. In Table 1, among the 12 cases of tamponade, except 1 bilateral case, 9 involved the left lung compared with 2 on the right.

**Table 1:** Previously reported cases of acute cardiac tamponade after pulmonary surgery

Case	Year	Excision	Time (postoperation)	Rescue way	Reason
**Morimoto M et al.** [4]	1991	RUL	13d	PCT	Aortic rupture at pericardium derivative
**Tovar EA et al.** [1]	1995	LLL	Intraoperative	PCT	Hemorrhage from the stump of the pulmonary vein in the pericardium
**McLean RH et al.** [2]	1999	RUL	5 h	PCT	Hemorrhage from the stump of the bronchial artery in the pericardium
**Jain et al.** [3]	2003	LUL	12 h	PCC	Hemorrhage from the stump of the pulmonary vein in the pericardium
**Tuinman AG et al.** [9]	2003	LP	2w	PCC (after sternotomy)	Unknown
**Neema PK** [5]	2011	LUL	3d	PCT	The pericardium injury
**Chen J et al.** [6]	2012	LUL	54 h	PCT	Pericardium and ventricle were injured
**Ozawa Y et al.** [8]	2013	wedge of RLL and left S8	4d	PCT	Coronary artery rupture
**Lee HM et al.** [10]	2017	LLL	1 h	PCT (PCC failure)	Unknown
**Astudillo MG et al.** [11]	2017	LLL	9d	PCC	Unknown
**Yamashita T et al.** [7]	2022	wedge of LLL	5d	PCC	The pericardium injury
**The case**		LUL	18 h	PCT (PCC recurrence)	Coronary artery rupture

RUL, right upper lobe. RLL, right lower lobe. LUL, left upper lobe. LLL, left lower lobe. LP, left pneumonectomy. PCC, pericardiocentesis. PCT, pericardiotomy

According to previous studies, the causes of coronary artery rupture included anatomical abnormalities, local infection, coronary intervention, coronary artery dissection and trauma [12]. Preoperative examination of our patient showed no abnormalities in the cardiovascular or cerebrovascular systems, and there was no relevant genetic history. However, the patient had a long history of diabetes with poor blood glucose control, resulting in long-term inflammatory stimulation and remodeling of the vascular wall [13]. Thorough inspection of the heart surface, pericardium, and coronaries revealed only the single site of coronary disruption. We postulated that the coronary artery rupture was caused by postoperative stress; accordingly it could be considered a case of spontaneous coronary rupture. We further speculated that the subsequent course of events may have been as follows: relief of the initial tamponade by pericardiocentesis allowed further hemorrhage from the coronary rupture, but recurrent tamponade compressed the vessel, and together with normal clotting, the hemorrhage was temporarily stopped. However, this closure was unstable, thrombus became dislodged, and with further hemorrhage, which led to more severe cardiac tamponade, necessitating pericardiotomy.

Acute cardiac tamponade is usually treated by pericardiocentesis or pericardiotomy, but the optimal surgical approach remains controversial [14]. In recent years, pericardiocentesis has become the preferred treatment after cardiac tamponade due to its advantages of less trauma, less risk, and the ability to be performed at the bedside. However, as shown in Table 1, of the 12 reported patients, 6 had an initial pericardiocentesis, but 1 failed and 1 recurred, both of which eventually underwent pericardiotomy. The details of the remaining patients were not reported in detail. Therefore, pericardiocentesis has certain limitations in patients with cardiac tamponade after lung surgery.

Although the incidence of acute cardiac tamponade following lobectomy is very low, its possibility, especially in conjunction with left lung surgery, should be considered and rapidly evaluated when the clinical situation suggests the diagnosis. Pericardiocentesis for acute tamponade is often appropriate as the immediate remedy, but the possibility of poor drainage or recurrence might necessitate a more definitive pericardiotomy.

### Electronic supplementary material

Below is the link to the electronic supplementary material.

Supplementary Material 1

Supplementary Material 2

## Data Availability

All data and materials in the case are available per request from the corresponding author on reasonable request.
