# Supplementary material for: Acute cardiac tamponade following thoracoscopic lobectomy: a case report and literatures review
Source: J Cardiothorac Surg. 2023 Oct 10;18:279. doi: 10.1186/s13019-023-02374-3 (PMC10563251; doi:10.1186/s13019-023-02374-3)
Supplement: Supplementary file 2 — Supplementary Material 2 [file 13019_2023_2374_MOESM2_ESM.docx]

**Author Information**

Wei Chen^1^, E-mail: 1270831632@qq.com

Yi Shen^1^, E-mail: 3503316244@qq.com

Yang Yuan^1^, E-mail: 329104274@qq.com

Qiangqiang Zheng^1^, E-mail: 1058541873@qq.com

Corresponding author: Yunfeng Zhou, E-mail: [yunf_zhou_hxsy@163.com](mailto:yunf_zhou_hxsy@163.com)

**Competing Interests**

None of the authors have potential competing interests with this manuscript.

Address: Department of Thoracic Surgery, West China School of Public Health and West China Fourth Hospital, Sichuan University, Chengdu, Sichuan, 610044, P.R. China.
